# Spatial Mapping of Glioblastoma Infiltration: Diffusion Tensor Imaging-Based Radiomics and Connectomics in Recurrence Prediction

**DOI:** 10.3390/brainsci15060576

**Published:** 2025-05-27

**Authors:** Kevin Jang, Michael Back

**Affiliations:** 1 Department of Radiation Oncology, Royal North Shore Hospital, Sydney, NSW 2065, Australia; kevin.jang@health.nsw.gov.au; 2 Faculty of Medicine and Health, The University of Sydney, Sydney, NSW 2050, Australia; 3 Genesis Care, Sydney, NSW 2015, Australia; 4 The Brain Cancer Group, Sydney, NSW 2065, Australia

**Keywords:** diffusion tensor imaging, glioblastoma, infiltration, recurrence

## Abstract

Glioblastoma (GBM) often exhibits distinct anatomical patterns of relapse after radiotherapy. Tumour cell migration along myelinated white matter tracts is a key driver of disease progression. The failure of conventional imaging to capture subclinical infiltration has driven interest in advanced imaging biomarkers capable of quantifying tumour–brain interactions. Diffusion tensor imaging (DTI), radiomics, and connectomics represent a triad of innovative, non-invasive approaches that map white matter architecture, predict recurrence risk, and inform biologically guided treatment strategies. This review examines the biological rationale and clinical applications of DTI-based metrics, radiomic signatures, and tractography-informed connectomics in GBM. We discuss the integration of these modalities into machine learning frameworks and radiotherapy/surgical planning, supported by landmark studies and multi-institutional data. The implications for personalised neuro-oncology are profound, marking a shift towards risk-adaptive, tract-aware treatment strategies that may improve local control and preserve neurocognitive function.

## 1. Introduction

High-grade gliomas are rapidly progressive, characterised by widespread infiltration into the brain parenchyma. Glioblastoma (GBM) is associated with the worst prognosis, with a median survival of 14–18 months despite maximal safe resection followed by adjuvant chemoradiotherapy [1,2]. The poor survival rates are largely attributable to tumour progression, with high rates of locoregional recurrence even after maximal resection and high-dose radiotherapy [2]. Accurate delineation of infiltrative margins remains difficult due to extensive tumour spread beyond the contrast-enhancing border [3,4,5]. Moreover, glioma cells are often resistant to chemo- and radiotherapy, driven by the profound intratumoural heterogeneity and a complex microenvironment [2,3]. Recurrent GBM frequently arises in remote, non-contiguous regions of the brain, often along established white matter tracts [6,7,8]. A greater understanding of tumour growth and infiltration is required to characterise the patterns of GBM recurrence and tailor treatment delivery to individual patients.

These recurrence patterns challenge the assumption that spatial proximity alone defines recurrence risk. Instead, the brain’s underlying structural connectivity appears to guide tumour cell migration [7]. The inability of conventional magnetic resonance imaging (MRI) to visualise microscopic infiltration underpins the need for more sensitive and biologically informed imaging biomarkers. In this context, diffusion tensor imaging (DTI), radiomics, and structural connectomics have emerged as valuable tools to quantify white matter disruption, characterise peritumoural heterogeneity, and model tumour–brain network interactions [9,10,11].

The biological behaviour of GBM necessitates a shift in imaging paradigms—from morphological to functional and microstructural characterisation. DTI, which captures directional water diffusion, provides valuable insight into white matter integrity [9]. When combined with radiomic analysis and tractography-informed connectomics, DTI offers a platform to quantify infiltration, predict recurrence, and guide radiotherapy/surgical planning. This review explores the biological rationale and translational utility of these imaging approaches in predicting GBM recurrence. We synthesise key findings from landmark studies, evaluate methodological innovations, and highlight future opportunities for integrating these tools into personalised neuro-oncology workflows.

## 2. White Matter Tracts and the Infiltrative Nature of Glioblastoma

GBM is characterised by diffuse invasion into the surrounding brain parenchyma. Unlike metastatic lesions that form discrete, well-demarcated nodules, glioblastoma exhibits a highly infiltrative phenotype, spreading insidiously along pre-existing anatomical structures—particularly myelinated white matter tracts [7]. This pattern of perivascular and periaxonal invasion enables tumour cells to migrate far beyond the radiologically apparent tumour margins, challenging conventional imaging thresholds and therapeutic boundaries [3,4,5,7].

The molecular underpinnings of this infiltrative behaviour involve a complex interplay between tumour cells and the extracellular matrix. Glioma cells exhibit upregulation of cell adhesion molecules, including integrins (notably αvβ3 and αvβ5), cadherins, and selectins, which facilitate adherence to white matter scaffolds [12]. Concurrently, the secretion of matrix metalloproteinases, such as MMP-2 and MMP-9, allows degradation of extracellular matrix components, paving the way for cellular migration [13]. Invasive front signalling through pathways such as PI3K/AKT, TGF-β, and Wnt further promotes motility and survival in the peritumoural microenvironment [14,15].

Seminal work by Giese et al. provided robust preclinical evidence for the directional migration of glioma cells along aligned white matter tracts, including the corpus callosum, internal capsule, and arcuate fasciculus [16]. Using rodent models, they demonstrated that glioma cells displayed preferential alignment with axonal bundles and avoided crossing grey matter boundaries. This finding was among the first to suggest that glioma cell invasion is not random but is constrained and facilitated by the anatomical architecture of the brain. Subsequent in vivo and ex vivo studies corroborated these findings in human specimens, showing that glioma cells can extend several centimetres beyond the enhancing lesion, even in the absence of radiological abnormality on conventional sequences such as T1-weighted or FLAIR MRI [4,5].

The clinical relevance of this anatomical preference was validated in a landmark study by Price et al., who employed image-guided biopsy to sample peritumoural tissue in patients with newly diagnosed GBM [17]. By mapping biopsy sites to DTI-derived fractional anisotropy (FA) maps, the authors demonstrated that areas of reduced FA correlated with histopathologically confirmed infiltration. Notably, many of these regions appeared morphologically normal on standard MRI. This study established DTI as a functional imaging biomarker capable of revealing subclinical disease—information that has direct implications for defining surgical margins and radiotherapy target volumes.

Patterns of recurrence further support the hypothesis that glioblastoma spreads preferentially along white matter tracts. Niyazi et al. observed that distant and contralateral recurrences were often aligned with midline structures, particularly the corpus callosum [18]. These findings suggest that tumour spread is not governed solely by spatial proximity to the original lesion, but by the connectivity of white matter pathways that act as biological conduits for cellular migration. Ellingson et al. extended this concept using functional diffusion maps (fDMs), demonstrating that early post-treatment changes in diffusion metrics, such as declining FA or increasing MD, were spatially predictive of future recurrence, often weeks before radiological progression became apparent [19].

Such findings challenge traditional models of tumour growth and underscore the limitations of isotropic margin expansion strategies in radiotherapy. The assumption that microscopic spread radiates uniformly from the tumour core does not hold in the presence of tract-guided invasion. Instead, tumour cells exploit the anisotropic geometry of the brain, selectively invading along established axonal pathways [9,16,17,18,19]. This has led to growing consensus that anatomical connectivity, rather than Euclidean distance, may be the dominant determinant of recurrence risk in GBM.

The implications for clinical management are substantial. By recognising the directional and tract-specific nature of glioblastoma infiltration, clinicians can move towards biologically informed treatment strategies. This includes asymmetric margin delineation during radiotherapy planning, prioritisation of tract-adjacent tissue for dose escalation, and integration of white matter tractography into surgical navigation systems [11,20,21,22]. Moreover, it highlights the potential for advanced imaging modalities, particularly DTI and tractography, to serve not just as diagnostic tools but as predictive instruments for recurrence and survival [9,19].

The infiltrative nature of glioblastoma is deeply rooted in the structural and functional organisation of the brain. Through its interaction with white matter tracts, the tumour transcends anatomical boundaries in ways that are invisible to conventional imaging but increasingly detectable through diffusion-based techniques. A thorough understanding of this behaviour is essential for advancing precision in imaging, planning, and ultimately, patient outcomes.

## 3. Diffusion Tensor Imaging: Principles, Metrics and Clinical Insights

DTI is a valuable MR technique that extends conventional diffusion-weighted imaging by capturing the directional movement of water molecules within tissue [23]. In the human brain, particularly in white matter, water diffusion is anisotropic due to the highly ordered alignment of axonal fibres and the restrictive properties of myelin. By modelling the diffusion process in three dimensions, DTI offers a unique window into the brain’s microstructural architecture and enables non-invasive characterisation of white matter integrity.

The core mathematical construct in DTI is the diffusion tensor, which characterises the variance of diffusion across spatial axes. From this tensor, several scalar metrics are derived, each reflecting specific aspects of tissue organisation. Fractional anisotropy quantifies the degree of directionality in diffusion and serves as a surrogate for axonal coherence and integrity. Mean diffusivity reflects the overall magnitude of water diffusion and increases in the presence of oedema or tissue breakdown [24]. Axial diffusivity (AD) and radial diffusivity (RD) further decompose the diffusion tensor to capture axonal and myelin integrity, respectively. More recently, the tract density index (TDI), derived from probabilistic tractography, has emerged as a means of quantifying fibre density on a voxel-wise basis, offering spatial resolution of white matter disruption [24]. A summary of DTI-based parameters can be found in Table 1.

In the context of glioblastoma, these DTI-derived measures have demonstrated considerable clinical relevance. Glioblastoma exhibits an infiltrative growth pattern, extending far beyond contrast-enhancing tumour margins into seemingly normal-appearing brain parenchyma. This microscopic invasion disrupts white matter microstructure in a manner that is not reliably captured by conventional MRI. Studies have shown that regions surrounding glioblastoma exhibit reductions in FA and elevations in MD and RD, reflecting fibre disorganisation, vasogenic oedema, and early tissue degradation. Importantly, these changes may precede or occur independently of radiological contrast enhancement [5,8].

Fractional anisotropy remains one of the most clinically informative DTI metrics for assessing glioblastoma infiltration and predicting recurrence. Histopathological validation by Price et al. demonstrated that regions of reduced FA, often indistinct on conventional MRI, corresponded with histologically confirmed tumour infiltration, supporting FA as a surrogate for microscopic disease spread [17]. Subsequent studies have expanded on these findings, showing that decreased FA in peritumoral or non-enhancing regions correlates with early recurrence, tumour progression, and poorer treatment response. Importantly, FA alterations have been detected weeks before radiographic evidence of recurrence emerges, suggesting potential for pre-emptive intervention [5,6,7,8,9].

Moreover, diffusion time and directional diffusivity analyses have revealed that recurrence often follows preferential pathways of least resistance (preserved white matter tracts) linking tumour spread with structural connectivity. Recognising that conventional DTI metrics can be confounded by peritumoral oedema, studies have applied free-water correction to isolate tissue-specific diffusion properties [25]. Using this approach, areas of eventual recurrence demonstrate significantly lower free-water-corrected FA and mean diffusivity values, enhancing the specificity of DTI for true tumour infiltration [25]. These findings reinforce the value of advanced DTI models in surgical and radiotherapeutic planning, offering earlier and more accurate delineation of high-risk regions that may otherwise be underestimated on anatomical imaging alone.

One limitation of conventional DTI parameters is the assumption of Gaussian diffusion, which may oversimplify the heterogeneous tumour microenvironment. **Diffusion kurtosis imaging (DKI)** extends this framework by quantifying the non-Gaussianity of water diffusion, offering deeper insights into tissue complexity. Emerging studies have shown that DKI parameters, particularly mean kurtosis and radial kurtosis, are sensitive to glioblastoma infiltration along white matter tracts, even in areas that appear normal on anatomical imaging. Pogosbekyan et al. demonstrated that gradients in DKI metrics reflected varying degrees of tumour cell density and invasion, with higher kurtosis values correlating with more aggressive infiltration and microstructural distortion [26]. These findings suggest that DKI may enhance detection of occult tumour spread, particularly in peritumoral or non-enhancing regions, and could serve as a valuable adjunct to DTI in delineating resection margins and identifying high-risk tissue during treatment planning.

The tract density index provides an additional perspective by quantifying the spatial density of reconstructed fibre pathways, effectively mapping white matter network integrity. Regions of low TDI adjacent to the tumour core have been correlated with improved overall survival [27]. These spatially resolved maps are particularly useful in guiding margin expansion during radiotherapy or informing resection strategies in eloquent brain regions where preservation of function is paramount.

DTI has also proven valuable in longitudinal imaging. Functional diffusion maps, developed by Ellingson et al., enable voxel-wise comparison of diffusion metrics over time and can identify areas of emerging recurrence or treatment resistance prior to radiological progression [19]. Their application has demonstrated that dynamic changes in FA and MD during radiotherapy are predictive of treatment response and progression-free survival. These findings point to a potential role for DTI not only in baseline imaging but also in adaptive treatment workflows that respond to evolving biological signals.

The integration of DTI into clinical neuro-oncology workflows is becoming increasingly feasible due to advances in acquisition protocols, standardisation of processing pipelines, and compatibility with treatment planning systems. Several commercial platforms now allow DTI datasets to be imported into contouring software, and semi-automated tractography workflows have streamlined the generation of tract density and diffusion maps [28]. Nonetheless, challenges remain, particularly with respect to susceptibility to artefacts, standardisation across institutions, and the interpretability of derived features in complex anatomical regions.

Despite these limitations, DTI offers a distinct and complementary dimension of tumour assessment that extends far beyond the capabilities of anatomical imaging. By visualising microstructural disruption at the tumour–brain interface, DTI provides a biologically grounded framework for identifying high-risk regions, predicting recurrence, and tailoring therapeutic strategies. Its utility spans the continuum of care, from preoperative planning to postoperative surveillance and radiotherapy guidance. As the field moves toward precision neuro-oncology, DTI is poised to become a foundational imaging modality in the management of glioblastoma.

## 4. Radiomics and Machine Learning in DTI-Based GBM Imaging

**Figure 1:**
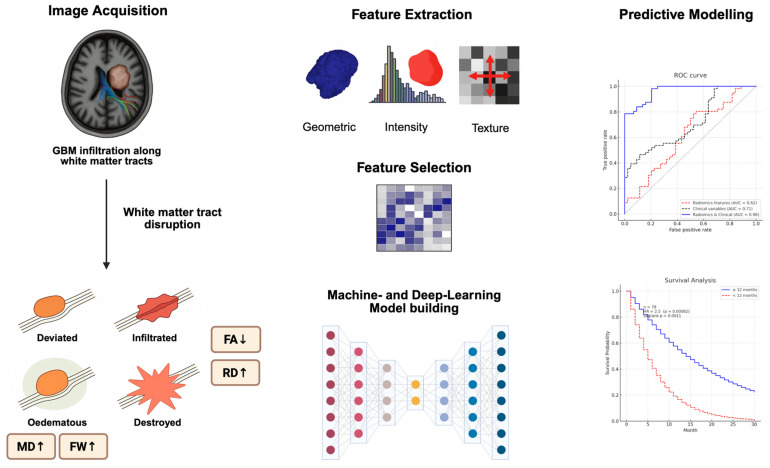
Schematic overview illustrating the integration of structural and diffusion imaging biomarkers in GBM. Tumour infiltration disrupts white matter tracts, which is reflected in altered DTI metrics (e.g., reduced FA, elevated MD, radial diffusivity (RD), and free-water fraction). Quantitative radiomic features (shape, intensity, and texture) are extracted from tumour and peritumoral regions. Following feature selection, machine learning and deep learning models are trained to predict clinical outcomes.

Radiomics refers to the high-throughput extraction of quantitative imaging features from radiological scans, encompassing shape, intensity, and texture descriptors [9]. When applied to DTI, radiomics captures heterogeneity in diffusion patterns that may signal infiltration, proliferation, or treatment resistance. By converting imaging data into high-dimensional feature sets, encompassing intensity, texture, shape, and spatial relationships, radiomics offers the potential to characterise tumours in ways that are inaccessible through visual interpretation alone [10]. Figure 1 provides a schematic overview of diffusion-based radiomics modelling of GBM infiltration. Common radiomic parameters can also be found in Table 2.

The microstructural sensitivity of DTI makes it particularly suited for radiomic analysis in glioblastoma. As tumour cells infiltrate surrounding white matter, they induce subtle changes in diffusion properties—manifesting as alterations in FA, MD, and other tensor-derived metrics. These variations are spatially heterogeneous, often extending beyond contrast-enhancing margins, and serve as surrogates for microscopic disease spread, tissue disruption, and response to therapy. Radiomics captures this complexity through textural features that quantify signal variation and spatial organisation, allowing clinicians to derive biologically relevant information from conventional imaging protocols.

A landmark study by Kim et al. exemplified the clinical utility of DTI-based radiomics in post-treatment assessment [46]. The authors extracted a suite of radiomic features from FA and MD maps and trained a machine learning classifier to differentiate pseudoprogression from true tumour progression—two entities that are radiologically indistinguishable but have vastly different management implications. Their model, which integrated grey-level co-occurrence matrix (GLCM)-based metrics such as entropy and dissimilarity, achieved an area under the curve (AUC) of 0.91. Importantly, the classifier outperformed both conventional imaging interpretation and standard clinical variables, highlighting the added value of DTI radiomics in complex diagnostic scenarios.

Li et al. expanded on this approach by combining radiomic features with clinical data in a support vector machine (SVM) framework to classify recurrence subtypes [47]. Their model incorporated features such as GLCM correlation, run-length nonuniformity, and histogram-based kurtosis derived from DTI maps. The resulting classifier achieved AUCs exceeding 0.86 across independent validation cohorts, demonstrating that diffusion-based radiomic signatures can meaningfully differentiate recurrence phenotypes and inform prognostic stratification.

In a similarly impactful study, Saksena et al. focused on the peritumoural zone, hypothesising that the tissue surrounding the tumour core contains critical information about tumour infiltration and biological behaviour [48]. By extracting radiomic features from treatment-naïve DTI scans, they demonstrated that texture heterogeneity in the peritumoural brain correlated significantly with progression-free and overall survival. These findings underscored the prognostic value of non-enhancing regions, which are often underemphasised in clinical practice but may harbour substantial infiltrative burden.

Beyond handcrafted features, recent efforts have focused on applying deep learning to raw DTI data. Convolutional neural networks (CNNs) have been trained to automatically learn relevant features from voxel-level diffusion maps, bypassing the need for manual feature engineering [49,50]. While these models require large, annotated datasets and are susceptible to overfitting, they offer significant advantages in automation, scalability, and potentially superior performance. Early studies have shown that CNNs trained on multiparametric DTI inputs can predict survival and recurrence with high accuracy, although further validation in multicentre cohorts is needed [49,50].

An emerging paradigm in glioblastoma imaging is radiogenomics, the integration of quantitative imaging features with genomic and molecular tumour profiles [10,51]. Radiogenomic studies have demonstrated that distinct molecular subtypes exhibit characteristic patterns in DTI-derived and texture-based features. For instance, IDH-mutant gliomas are typically associated with higher fractional anisotropy, greater tract coherence, and preserved white matter architecture, reflecting their more indolent growth and limited infiltration. In contrast, IDH-wild-type GBMs often display lower FA, reduced tract integrity, and elevated texture heterogeneity, particularly in peritumoral regions, suggesting more aggressive infiltration and disrupted microstructure [10,51]. These imaging phenotypes correlate with tumour biology and clinical outcomes, enabling non-invasive stratification of disease aggressiveness.

Beyond IDH status, EGFR-amplified tumours have been linked to increased directional diffusivity, higher kurtosis, and marked entropy changes on DTI-based radiomics, consistent with their known proliferative and invasive behaviour. Such associations open avenues for non-invasive molecular inference: several studies have shown that radiogenomic models can predict MGMT methylation, mesenchymal transition, and other transcriptional signatures from diffusion- and texture-based features alone [10,51]. This capability holds significant clinical promise, not only in reducing the reliance on repeat biopsy but also in identifying spatially heterogeneous subclones, guiding biopsy targeting, and informing precision radiotherapy strategies such as focal dose escalation or margin adaptation. As computational models become more sophisticated and validated in multicentre cohorts, radiogenomics stands poised to become an integral tool in personalised glioblastoma management.

The application of machine learning in this context has required careful attention to algorithm selection, feature reduction, and cross-validation [52]. Feature selection techniques such as LASSO regression, principal component analysis, and recursive feature elimination are commonly employed to avoid overfitting and improve generalisability. Models are often validated using k-fold cross-validation, bootstrapping, or independent external cohorts—an essential step for clinical translation [10,51,52].

Despite the considerable promise of DTI-based radiomics and machine learning, several challenges remain. Standardisation of acquisition parameters and preprocessing workflows is critical to ensure reproducibility across institutions. Differences in scanner hardware, voxel resolution, and segmentation protocols can introduce variability that affects model performance. Moreover, interpretability remains a key barrier to clinical adoption; while many radiomic models achieve high accuracy, their decision-making processes are often opaque, limiting clinician trust [10,53].

Nevertheless, the convergence of DTI radiomics and machine learning represents a significant leap toward precision imaging in neuro-oncology. By quantifying tissue heterogeneity and leveraging computational intelligence, these tools provide clinicians with powerful, non-invasive biomarkers for risk stratification, treatment response, and recurrence prediction. As larger, harmonised datasets become available and as regulatory frameworks for AI in healthcare mature, DTI-based radiomics is likely to become an integral component of the neuro-oncological armamentarium.

## 5. Structural Connectomics and Probabilistic Tractography

Connectomics is the study of the brain’s network architecture, constructed from DTI-derived white matter pathways and graph theoretical models [54,55,56]. Glioblastoma’s interaction with the structural connectome is not incidental but biologically deterministic. Tumour cells migrate preferentially along myelinated white matter pathways, using these tracts as low-resistance conduits for invasion. As infiltration progresses, the structural network becomes increasingly compromised, manifesting as a degradation of topological features such as global efficiency, nodal strength, and betweenness centrality [22]. These disruptions can be quantitatively assessed through graph theoretical analysis of DTI-derived tractography, providing a systems-level view of tumour impact that transcends anatomical localisation [54]. Recent progress in the clinical applications of connectomics and radiomics in GBM can be found in Table 3.

Liu et al. provided compelling evidence of this phenomenon by constructing individualised structural connectomes from preoperative DTI data in patients with newly diagnosed glioblastoma [22]. Their analysis revealed that nodes with high centrality, especially those located within default mode and salience networks, were disproportionately affected by tumour infiltration. Reduced nodal efficiency and compromised hub integrity were predictive of distant recurrence and poorer survival. These findings underscore the principle that tumour spread is not random but guided by network topology, with high-connectivity regions acting as attractors for secondary invasion.

The prognostic relevance of global network disruption was further validated by Derks et al., who demonstrated that reduced global efficiency within the structural connectome independently correlated with shorter overall survival [58]. Even after adjusting for conventional prognostic markers such as tumour volume and resection extent, network integrity remained a significant predictor of outcome. Importantly, these disruptions were observed not only in regions directly affected by tumour but also in distant nodes, suggesting that glioblastoma exerts a system-wide effect on brain connectivity.

Probabilistic tractography, a technique that reconstructs fibre pathways from DTI data, offers a means of visualising and quantifying these disruptions with greater anatomical specificity [57]. Unlike deterministic tractography, which assigns a single trajectory to each diffusion vector, probabilistic approaches account for the inherent uncertainty in diffusion orientation—particularly in areas with crossing fibres or distortion from tumour mass effect. This makes probabilistic tractography more robust in the context of glioblastoma, where peritumoural architecture is often highly distorted.

Clinically, tractography has been used to identify white matter tracts at risk of tumour infiltration or involved in functional processing. Tracts such as the corticospinal tract, arcuate fasciculus, inferior fronto-occipital fasciculus, and corpus callosum are frequently implicated due to their proximity to common tumour locations. Studies have demonstrated that regions of tract disruption, as visualised through FA loss or reduced streamline density, often align with eventual recurrence zones, even in areas that appear structurally intact on conventional imaging [27].

Jena et al. pioneered the integration of DTI-based tractography into radiotherapy planning [20]. In their study, functional white matter tracts were delineated using DTI and used to guide margin expansion asymmetrically targeting high-risk regions along fibre tracts while preserving eloquent structures. This strategy not only improved the coverage of subclinical disease but also reduced the irradiation of functionally critical tissue. Their work marked an early step toward biologically informed, tract-aware radiotherapy design.

More recently, tractography has been employed to inform surgical navigation, enabling neurosurgeons to preserve vital white matter pathways during resection. Preoperative mapping of the corticospinal tract, arcuate fasciculus, and optic radiation has improved postoperative functional outcomes, particularly when integrated with intraoperative neuro-monitoring. This has been especially valuable in cases of tumours in eloquent or deep-seated locations, where complete resection must be balanced against preservation of neurological function [21,64].

Preoperative tractography has proven especially beneficial for GBM located near motor or language pathways. Qiu et al. demonstrated that integrating stereoscopic 3D visualisations of DTI tractography with structural MRI enabled precise mapping of tumours relative to the corticospinal tract, enhancing surgical trajectory selection and minimising postoperative deficits [64]. Such planning is not only valuable for visualising tract displacement or compression but also for stratifying resection risk based on proximity and anisotropy changes. Wu et al. further validated this in a prospective study comparing DTI-guided and conventional neuronavigation, showing significantly higher gross total resection rates and lower incidence of motor deficits in the DTI group [21]. These findings highlight that the incorporation of diffusion metrics into surgical planning does not merely refine anatomical detail but directly improves clinical outcomes.

When co-registered with contrast-enhanced MRI in neuronavigation systems, DTI tractography offers real-time intraoperative reference for navigating eloquent white matter tracts. Bello et al. reported that preoperative tractography changed surgical strategy in over a quarter of glioma cases, influencing craniotomy placement, resection planes, and surgical extent [60]. The integration of these modalities has achieved sub-millimetre accuracy, allowing surgeons to visualise tract involvement dynamically, even as anatomy is distorted intraoperatively. This tract-aware approach has been particularly effective in preserving pyramidal and language function during resections in high-risk zones. The growing body of evidence suggests that the use of DTI in surgical planning should not be reserved for complex cases alone but considered standard practice in functionally constrained glioma resections.

Beyond surgical and radiotherapeutic applications, connectomic analysis may also enhance patient stratification and longitudinal monitoring. By quantifying the trajectory of network disruption over time, clinicians may be able to predict cognitive decline, therapeutic response, or risk of recurrence with greater precision. Preliminary studies have suggested that changes in connectomic metrics during chemoradiotherapy correlate with treatment response, although further validation in prospective cohorts is needed [57].

Despite these promising advances, several challenges must be addressed before structural connectomics and tractography can be fully integrated into routine clinical workflows. Variability in tractography algorithms, lack of standardised parcellation schemes, and susceptibility to artefacts remain barriers to reproducibility. Furthermore, the computational complexity of constructing and analysing individualised connectomes has historically limited their use to research settings. However, recent advances in automated segmentation tools, such as those provided by MRtrix3 and TractoFlow, have significantly reduced the processing burden, bringing connectomics closer to clinical feasibility [28,63].

In summary, structural connectomics and probabilistic tractography are valuable tools to understand glioblastoma invasion and its clinical consequences. By mapping the architecture of brain networks and quantifying their disruption, these tools offer unique insights into tumour biology, recurrence risk, and therapeutic vulnerability. As methods become more standardised and clinically accessible, their integration into neuro-oncology promises to enhance both diagnostic precision and treatment personalisation.

## 6. Future Directions and Clinical Translation

The integration of advanced neuroimaging techniques into the management of glioblastoma represents a paradigm shift from morphologically based decision-making to biologically informed precision medicine. Yet, despite the promise demonstrated across multiple studies, the widespread clinical adoption of these tools remains limited. As the field matures, bridging this translational gap will require coordinated advances in imaging standardisation, data infrastructure, machine learning integration, and clinical trial design [53]. Table 4 summarises the key translational barriers and their proposed solutions.

A major challenge is the harmonisation of DTI acquisition protocols across institutions. Variability in MRI scanner platforms, b-values, number of diffusion directions, and voxel resolution introduces significant heterogeneity in DTI outputs. These inconsistencies hinder reproducibility and limit the generalisability of radiomic and connectomic models. Initiatives such as the Quantitative Imaging Biomarkers Alliance (QIBA) and the GLASS consortium are actively working to develop consensus protocols, phantom calibration standards, and cross-platform correction methods [61,66]. Adoption of such standardisation frameworks is essential to support multicentre trials and regulatory validation.

Computational reproducibility must also be addressed. Radiomic features and tractography outputs are sensitive not only to acquisition but also to preprocessing steps, including image resampling, segmentation, and denoising. Recent open-source pipelines, such as TractoFlow and MRtrix3, offer containerised workflows that improve consistency and transparency [28,63]. Additionally, feature extraction tools like PyRadiomics and Dipy now include reproducibility testing modules that can be incorporated into research pipelines and, eventually, clinical software [59,67].

The next frontier lies in clinical-grade machine learning integration. Numerous studies have demonstrated the power of machine learning models in predicting recurrence, classifying progression, and estimating survival using DTI-derived features [48]. However, few of these models have progressed beyond retrospective research environments. Clinical deployment requires algorithm interpretability, prospective validation, and seamless integration into radiology and radiotherapy information systems. Explainable AI (XAI) techniques—including saliency mapping and SHAP values, may offer pathways to demystify decision boundaries and build clinician trust [59,67].

To accelerate clinical implementation, multiparametric fusion of imaging modalities will become increasingly important. The combination of DTI with amino acid PET (e.g., FET-PET), MR spectroscopy, and perfusion-weighted imaging can yield a more holistic view of tumour biology. For instance, integrating PET-derived metabolic activity with DTI-based tract disruption may identify spatially distinct biological targets for dose painting. Such imaging fusion strategies are currently being explored in the context of biologically adaptive radiotherapy, where dose is modulated based on multimodal imaging biomarkers over the course of treatment [68,69].

Radiogenomics represents another compelling area of development. Radiomic features extracted from DTI and other imaging sequences have been shown to correlate with glioblastoma molecular subtypes, including IDH mutation, MGMT methylation, and EGFR amplification [70]. These associations suggest that imaging can serve as a non-invasive surrogate for genomic profiling, guiding treatment intensification or de-escalation when tissue sampling is infeasible. Large-scale radiogenomic datasets, such as TCIA and TCGA-GBM, are facilitating the development of such models, though prospective validation in diverse populations remains necessary [71].

A transformative concept on the horizon is longitudinal connectome monitoring. By mapping how glioblastoma alters brain network topology over time—either as a consequence of disease progression or treatment-induced neurotoxicity—clinicians may be able to predict not only recurrence but also cognitive decline and functional outcomes. Connectomic markers such as global efficiency, modularity, and hub disruption may become endpoints in neuro-oncology trials, guiding therapeutic decisions that balance survival benefit with quality-of-life preservation [58].

To facilitate these innovations, prospective clinical trials must be designed with imaging biomarker endpoints. For example, trials could randomise patients to receive either standard radiotherapy or DTI-informed, tract-aware planning to evaluate differences in recurrence patterns, neurocognitive preservation, and survival. Imaging biomarkers should also be incorporated into adaptive trial frameworks, where radiomic and connectomic risk models inform treatment intensification strategies. The use of federated learning platforms—allowing AI models to be trained across institutions without sharing patient data—offers a scalable and privacy-conscious mechanism for building robust predictive algorithms [70,72].

Finally, regulatory and implementation science must be engaged to support translation. Imaging biomarkers and AI models must meet standards for medical device approval, with attention to robustness, reproducibility, and patient safety. Clinical implementation will also require training clinicians to interpret novel biomarkers, restructuring radiotherapy workflows to accommodate tractography and voxel-based planning, and demonstrating cost-effectiveness in real-world health systems [10].

In summary, the convergence of DTI, radiomics, and connectomics in GBM offers a transformative path forward—one where imaging is no longer static or anatomical, but dynamic, networked, and biologically expressive. While challenges remain, the technological and scientific foundations are now in place. With investment in standardisation, validation, and workflow integration, these tools are poised to reshape how glioblastoma is imaged, treated, and understood. The future of neuro-oncology will not be defined by tumour size alone, but by its spatial biology, network disruption, and molecular phenotype—all mapped through the lens of advanced imaging.

## 7. Conclusions

The integration of DTI, radiomics, and connectomics into the management of glioblastoma represents a shift from anatomical to biological imaging. These modalities offer unprecedented insight into tumour behaviour, white matter infiltration, and recurrence risk. When embedded into machine learning frameworks and radiotherapy planning systems, they enable tract-aware, risk-adaptive treatment strategies that have the potential to improve local control while preserving function. As technical barriers are addressed and validation accrues, these tools will transition from academic innovation to clinical standard, transforming neuro-oncology into a precision discipline guided by the invisible architecture of the brain.

## Figures and Tables

**Table 1:** DTI-based quantitative parameters.

Parameter	Definition
Fractional Anistropy (FA)	Scalar value (0–1) representing the degree of directional diffusion of water molecules.
Mean Diffusivity (MD)	Average magnitude of diffusion regardless of direction (in mm^2^/s).
Axial Diffusivity (AD)	Diffusivity parallel to the principal eigenvector of diffusion.
Radial Diffusivity (RD)	Diffusivity perpendicular to the principal fibre direction.
Tract Density Index (TDI)	Quantifies the number of streamlines (fibres) per voxel from probabilistic tractography.
Anisotropy Coefficient Maps	Maps derived from FA distribution to visualise fibre orientation and coherence.

**Table 2:** Radiomic parameters used in brain tumour imaging.

Parameters and Main References	Definition
***First-order texture statistics***	
Entropy [29]	Measures the inherent randomness in the grey-level intensities of an image or ROI.
Uniformity [29]	Measures the homogeneity of grey-level intensities within an image or ROI.
***Higher-order texture statistics***	
Grey-level co-occurrence matrix [30]	Examines the spatial distribution of grey-level intensities within an image through a 2D grey tone histogram.
Angular second movement [29]	Measures the textural uniformity of an image (also referred to as homogeneity). Captures the two-dimensional complexity of the edge of the tumour abnormalities.
Inverse difference moment [29]	Measures local image homogeneity as it assumes larger values for smaller grey tone differences in pair elements.
Contrast [29]	Measures the spatial tone frequency of an image as the difference between the highest and lowest values of a contiguous set of pixels.
Correlation [29]	Measure of grey tone linear dependencies in the image.
Bounding ellipsoid volume ratio [31]	Ratio of the tumour volume to the volume of the smallest ellipsoid that entirely encapsulates the tumour. Captures the three-dimensional complexity of tumours.
Semi-axis diameter ratios [32]	Ratios of the minor semi-axis length to the longest bounding ellipsoid semi-axis diameter. Captures the three-dimensional complexity of tumours.
Margin fluctuation [31,32]	Captures the two-dimensional complexity of the edge of the tumour abnormalities. Standard deviation of the difference between the ordered radial distances of the tumour edge from the centroid to all the boundary points, smoothed with an averaging filter of length equal to 10% of the tumour boundary.
Mean intensity [33]	Average intensity of the pixel values within the ROI.
Mean of positive pixel values [33]	Average pixel values of only the positive pixel values within the ROI.
Standard deviation (SD) [33]	Quantification of the variance from the mean value (high SD indicating wide variation in pixel values).
Kurtosis [33]	Peakedness (or pointedness) of the histogram of pixel values. Positive kurtosis = more peaked distribution. Negative kurtosis = flatter distribution.
Skewness [33]	Quantifies the asymmetry of the histogram. Negative skewness = longer tail on the left side of the histogram. Positive skewness = longer tail on the right.
Grey-level run matrix (GLRL) [34]	Number of contiguous voxels that have the same grey-level value. Characterises the grey-level run lengths of different grey-level intensities in any direction.
Short runs emphasis (SRE) [34]	Measures distributions of short runs. Higher values indicate fine textures.
Long runs emphasis (LRE) [34]	Measures distribution of long runs. Higher values indicate course textures.
Grey-level nonuniformity (GLN) [34]	Measures the distribution of runs over the grey values. Low value when runs are equally distributed along grey levels. A lower value indicates higher similarity in intensity values.
Run-length nonuniformity (RLN) [34]	Measures the distribution of runs over run lengths. Low value when runs are equally distributed over run lengths.
Run percentage (RP) [34]	Measures the fraction of the number of realised runs and the maximum number of potential runs. Highly uniform ROI volumes produce a low run percentage.
Neighbourhood grey tone difference matrix [35]	One dimensional matrix where each grey-level entry is the summation of the differences between all the pixels with grey-level value and the average grey-level value of its neighbourhood.
Coarseness [35]	Quantitative measure of local uniformity.
Busyness [35]	Rapid intensity changes of neighbourhoods in a given ROI.
Complexity [35]	Quantifies the complexity of the spatial information present in an image.
Texture strength [35]	Characterises the visual aesthetics of an image.
Local binary pattern (LBP) [36]	Quantifies local pixel structures through a binary coding scheme. Measures the tumour microenvironment.
Scale-invariant feature transform (SIFT) [36,37]	Detects distributed key points with a radius on tumour images. Measures tumour spatial characteristics.
Histogram of oriented gradients (HOG) [38]	Computes block-wise histogram gradients with multiple orientations. Measures the tumour microenvironment.
***Fractal***	
Fractal dimension (box-counting and sand-box algorithms) [39,40,41]	A non-integer number between 0 and 2, in a two-dimensional space, or 0 and 3, in a three-dimensional volume, that quantifies the space-filling properties of irregularly shaped objects.
Outline box dimension [29]	Evaluates the irregularity in shape of the image (i.e., how much it deviates from classic geometric figures).
Lacunarity [42]	Pixel distribution of an image at different box sizes and at various grid orientations. Describes the degree of non-homogeneity within an image.
***Spatial filtering***	
Median filter [43]	Reduces sparse noise. Sets each pixel in ROI equal to the median pixel value of its specified neighbourhood.
Entropy filter [44]	Accentuates edges by brightening pixels which have dissimilar neighbours. Sets each pixel in the ROI equal to the entropy (measure of disorder) of the pixel values in its specified neighbourhood.
Laplacian of Gaussian (LoG) filter [45]	The Laplacian filter is a derivative filter used to find areas of rapid change (edges) in an image. Images are first smoothed using a Gaussian filter before applying the Laplacian.

Reprinted with permission from ref. [10].

**Table 3:** Major developments in DTI-based quantitative imaging in GBM infiltration.

Author (Year)	Major Findings
Basser et al. (1994) [23]	Introduced the diffusion tensor formalism, establishing FA and MD as quantitative indices of white matter microstructure.
Pierpaoli et al. (1996) [24]	First human DTI study that mapped normal FA/MD distributions, providing the baseline against which tumour-related changes are measured.
Giese et al. (1996) [16]	Preclinical assays showed glioma cells migrate preferentially on myelinated substrates, inspiring tract-based imaging investigations.
Jena et al. (2005) [20]	Demonstrated anisotropic, tract-aware CTV expansion in radiotherapy planning using DTI, improving coverage while sparing eloquent tracts.
Sporns et al. (2005) [55]	Coined the “human connectome”, providing the graph-theory framework later applied to glioma network disruption.
Price et al. (2006) [17]	Image-guided biopsies proved reduced FA marks microscopic GBM infiltration beyond contrast enhancement.
Behrens et al. (2007) [57]	Developed probabilistic tractography accommodating crossing fibres—now standard for peritumoural mapping.
Wu et al. (2007) [21]	Prospective trial showed DTI-based neuronavigation increased safe resection and preserved motor outcomes.
Ellingson et al. (2012) [19]	Functional-diffusion-map changes during chemoradiotherapy predicted progression-free and overall survival weeks before MRI relapse.
Derks et al. (2014) [58]	Connectomic analysis revealed that decreased global efficiency and hub disruption correlate with survival in glioma patients.
Garyfallidis et al. (2014) [59]	Released DIPY, an open library for reproducible diffusion-MRI processing and tractography.
Bello et al. (2014) [60]	Combined tractography with intraoperative mapping to preserve language while maximising glioma resection.
Yu et al. (2016) [61]	Showed higher-grade gliomas produce greater reductions in connectome efficiency and modularity than lower-grade ones.
Saksena et al. (2010) [48]	Peritumoural DTI-texture heterogeneity independently predicted overall and progression-free survival.
Boss et al. (2024) [62]	QIBA multicentre phantom study quantified inter-scanner variability, establishing benchmark standards for DTI harmonisation.
Kim et al. (2019) [46]	DTI + perfusion radiomics model differentiated pseudoprogression from true progression with AUC 0.91.
Tournier et al. (2019) [28]	Published MRtrix3, a flexible open-source platform for advanced tractography and connectome construction.
Salvalaggio et al. (2023) [27]	Low peritumoural TDI values predicted improved overall survival.
Theaud et al. (2020) [63]	Launched TractoFlow, a containerised diffusion-MRI pipeline that standardises tractography across centres.
Yan et al. (2021) [49]	Deep learning features from whole-brain DTI stratified glioma risk groups and linked imaging phenotypes to molecular pathways.
Liu et al. (2024) [22]	Patient-specific connectomes showed hub disruption in default-mode/salience networks predicts distant recurrence and survival.
Wei et al. (2023) [11]	Structural connectome quantification of invasion provided an independent prognostic marker of overall survival in GBM.
Li et al. (2023) [47]	SVM radiomics model based on DTI distinguished GBM recurrence from radiation necrosis with multicentre AUC > 0.86.

**Table 4:** Barriers for clinical translation and proposed solutions.

Domain	Technical Barrier	Practical Solutions and Ongoing Initiatives
DTI acquisition and preprocessing	Scanner- and protocol-dependent variations in *b*-values, gradient directions, field strengths, and EPI distortion.	QIBA diffusion phantoms, vendor-neutral harmonised protocols, reverse-phase-encoded volumes for distortion correction, and site-wise ComBat harmonisation of diffusion metrics.
Radiomics feature stability	Handcrafted texture/shape features sensitive to voxel size, interpolation, and intensity discretisation.	IBSI-conformant feature definitions, resampling to isotropic voxels, test–retest repeatability (ICC > 0.85), and robust feature selection.
Machine learning interpretability	“Black-box” perception limits clinical trust.	Embedded XAI toolkits (SHAP, Grad-CAM) in PACS/RT-TPS viewers, model-agnostic partial-dependence plots, and calibration and decision-curve reporting [65].
Structural connectomics	No consensus on brain parcellation or tractography parameters, variable graph metrics.	Multi-atlas consensus parcellations (e.g., HCP-MMP 1.0), containerised pipelines (MRtrix3 Connectome, TractoFlow) with fixed seeds/thresholds, open-source code, and parameter disclosure.
Inter-institutional data sharing	Privacy laws restrict transfer of imaging/genomic data for external validation.	Federated learning frameworks (Flower, NVIDIA FLARE), differential-privacy aggregation, and synthetic-data augmentation.
Clinical workflow integration	Additional processing steps and limited DICOM-RT support hinder routine use.	Vendor plug-ins that auto-import parametric maps/tractograms into TPS, one-click containerised scripts, and automated QC and PDF summaries.
Regulation and reimbursement	AI software must demonstrate robustness, safety, and cost-effectiveness.	FDA/EMA SaMD guidance adherence, multicentre external validation, and health-economic models showing reduced recurrence and cognitive toxicity.

## Data Availability

No new data were created or analysed in this study.

## Abbreviations

AD: axial diffusivity; AI: artificial intelligence; ComBat: “combining batches” harmonisation; DICOM-RT: Digital Imaging and Communications in Medicine for Radiotherapy; DTI: diffusion tensor imaging; EPI: echo-planar imaging; FA: fractional anisotropy; FDA: US Food and Drug Administration; FLARE: Federated Learning Application Runtime Environment; HCP-MMP: Human Connectome Project Multimodal Parcellation; ICC: intraclass correlation coefficient; IBSI: Image Biomarker Standardisation Initiative; ML: machine learning; PACS: picture archiving and communication system; QIBA: Quantitative Imaging Biomarkers Alliance; QC: quality control; RD: radial diffusivity; RT-TPS: radiotherapy treatment planning system; SaMD: software as a medical device; SHAP: SHapley Additive exPlanations; TPS: treatment planning system; XAI: explainable artificial intelligence.
